# Benefits of Using Molecular Structure and Abundance in Phylogenomic Analysis

**DOI:** 10.3389/fgene.2012.00172

**Published:** 2012-09-06

**Authors:** Gustavo Caetano-Anollés, Arshan Nasir

**Affiliations:** ^1^Evolutionary Bioinformatics Laboratory, Department of Crop Sciences, University of IllinoisUrbana-Champaign, IL, USA

**Figure 1:**
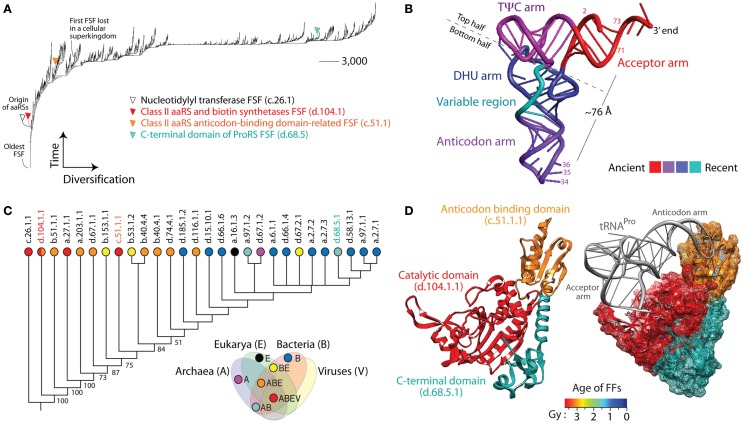
**Trees of domains (ToDs), tRNAs, and aminoacyl-tRNA synthetase (aaRS) history**. **(A)** ToD reconstructed from a genomic census of protein domain structures at fold superfamily (FSF) level of SCOP in 1,037 cellular organisms and viruses (Nasir et al., 2012). Taxa are FSFs and characters are proteomes. While leafs are not labeled with names of FSFs as these would not be legible, FSFs linked to selected domains of aaRS enzymes are identified. **(B)** tRNA molecule with arms colored according to their age (Sun and Caetano-Anollés, 2008). The most important identity determinants recognized by aaRSs are located in the anticodon loop of the anticodon arm and in the acceptor stem (labeled with nucleotide numbers), which are separated in space by ∼76 Å. **(C)** ToD describing the evolution of 28 domain families of aaRSs in 1,037 cellular organisms and viruses. Taxa are aaRSs fold families (FFs) and characters are proteomes. Numbers on the branches indicate bootstrap support values and taxa are labeled with SCOP *concise classification strings* with corresponding circles colored according to the distribution of families in cellular superkingdoms, Archaea (A), Bacteria (B), and Eukarya (E), and in viruses (V; e.g., families present in viruses are in red and correspond to the taxonomical group ABEV of the Venn diagram). Note that the catalytic domain of class II aaRSs, d.104.1.1, has been recently identified in megavirus but was absent in our dataset. **(D)** The prolyl-tRNA synthetase (ProRS) enzyme in complex with tRNA^Pro^ [PDB entry 1NJ8 from the archaeon *Methanocaldococcus janaschii* (left) and 1H4S from the bacterial extremophile *Thermus thermophilus* (right)] with its three domains (catalytic, anticodon-binding, and C-terminal) colored according to their age of origin (in billions of years, Gy). Domain ages were derived from a ToD at family level of structural complexity and ages placed in a geological timescale using the molecular clock of folds (Wang et al., 2011). Note how the variable arm of tRNA makes crucial contact with the anticodon-binding domain, both of which are evolutionarily derived, while the acceptor arm contacts the catalytic domain, both of which are ancient. This vividly illustrates tRNA and aaRS co-evolution.

Molecular structure is eminently modular and expresses complexity at different levels of molecular organization (Caetano-Anollés et al., 2009). At high levels, evolutionary change occurs at extraordinary slow pace. A new protein fold can take millions of years to materialize in sequence space while new sequences develop in less than microseconds. Structural cores are generally orders of magnitude more conserved than sequences. Consequently, they carry durable phylogenetic information useful for deep exploration of biological history. Unfortunately, the complexities of structural alignments, in which similarities of two sets of atoms with unknown correspondences are sought with no restriction on the correspondences, make global phylogenetic analysis of structure an enormous bioinformational challenge (Taylor, 2007). In recent years, however, a shift of focus from molecules to molecular repertoires, advances in bioinformatics implementations, and an expanded census of structure and function provided new avenues of evolutionary exploration. Developments include: (i) the almost complete experimental acquisition of protein folds structures (∼1,200 out of 1,500 expected; Levitt, 2009) and wide coverage of the modern RNA world (Leontis et al., 2006); (ii) functional ontologies with the potential to unify biological knowledge [e.g., gene ontology, (GO); Ashburner et al., 2000]; (iii) widespread and robust assignment of known structures to genomic sequences (Chothia and Gough, 2009); and (iv) the development of phylogenomic methods that embed structure and function directly into phylogenetic analysis (Caetano-Anollés et al., 2009). Genomic abundances derived from structural and functional censuses have been used to build trees of proteomes (ToPs; Gerstein, 1998), trees of domains (ToDs; Caetano-Anollés and Caetano-Anollés, 2003), and trees of functions (ToFs; Kim and Caetano-Anollés, 2010). While the branches of ToPs encase proteomic history and resemble traditional “trees of species” built by systematic biologists, ToDs describe how components of the system (domains in proteomes) change as the entire system evolves. These rooted phylogenomic trees establish an “evolutionary arrow,” without resorting to outgroup hypotheses, defining a chronology of architectural innovation (Figure 1A). Trees are not phenetic statements. While they are built from multistate or quantitative valued characters, speciation in trees fulfills a molecular clock that is compatible with paleobiology and the geological record (Wang et al., 2011). In sharp contrast to standard phylogenetic methods that generate trees of genes and genomes (ToGs) from the occurrence of genomic features (e.g., nucleotides or amino acid residues in sequence sites, presence/absence of a gene), ToDs and ToPs reap the benefit of processes occurring at higher and more conserved levels of the structural hierarchy that are responsible for the accumulation of modules in biology (Caetano-Anollés et al., 2010; Mittenthal et al., 2012). The systematic study of “abundance” of molecular parts rather than their “occurrence” offers several advantages over ToGs and standard phylogenetic analysis of sequence that we here highlight:

(1) ToDs and ToPs are derived from non-parametric models of genomic abundance that are free from problems of homology in the alignments of sequences and structures (Anisimova et al., 2010). Once structural and functional considerations assign a protein sequence to a domain structure (Murzin et al., 1995), homology is established. In contrast, sequence alignment remains problematic because there is still not an objective function in bioinformatics that can describe homology in sequence (especially, remote homology; Morrison, 2009). Sequence-based phylogenetic reconstruction relies by default on good multiple sequence alignments. However, alignment is a difficult bioinformatics and biological problem seeking to find similarities of two sets of sequences with unknown correspondences but restricted by the lineal order of residues. Without an objective and biologically inspired function to optimize, alignment remains the “weakest link” of phylogenetic analysis of sequences. This is aggravated by increases in error that are expected with increases in sequence divergence. Moreover, despite the development of number of structure-based alignment methods (Holm and Sander, 1993; Shindyalov and Bourne, 1998; Holm and Park, 2000; Koehl, 2001), finding an optimal and biologically significant alignment between two structures remains a difficult problem (Kolodny et al., 2005). In contrast, phylogenomic approaches that utilize abundance counts of protein domains as phylogenomic characters do not require computation of an alignment.
(2) ToDs and ToPs are not affected by the serious problem raised by Maddison (1993) of characters that are not applicable to all taxa in a data set, such as insertion/deletion (indel) sites. This problem plagues phylogenetic analysis of sequences (De Laet, 2005) and require of models that consider processes of indel generation (e.g., molecular growth and accretion), which are incipient.
(3) ToDs are highly imbalanced and do not follow the uniform (Yule) and random speciation models (Wang and Caetano-Anollés, 2009; Wang et al., 2011). ToPs are moderately imbalanced. Since tree imbalance occurs naturally when speciation depends on an evolving “heritable” trait (Heard, 1996), these patterns are expected outcomes from the accumulation of domains in proteomes (a biological process; Wang et al., 2011). In fact, increases in domain abundance are linked to increases of genes in genomes, which follow a Benford distribution that is persistent in evolution as the systems attempts to maximize information transmission (Friar et al., 2012). In contrast, ToGs show moderate imbalance (Herrada et al., 2011), which can be either expression of semipunctuation during speciation, high extinction rates, or an artifact resulting from heterotachy (Venditti and Pagel, 2010). Separating these possible culprits is difficult and needed before assigning value to individual phylogenies. While different evolutionary models have been proposed for branching patterns in ToGs (Mooers and Heard, 1998), especially those describing “trees of species,” scaling properties at gene and species level must be sought and their universality tested in order to understand branching rules (Herrada et al., 2011).
(4) Taxon sampling represents a problem in phylogenetic analysis that impacts accuracy and phylogenetic error (Zwickl and Hillis, 2002). ToDs are refractory to the problem since they sample the set of all known domains (i.e., they portray history of an operationally finite set of taxa).
(5) The troublesome task of solving the problem of orthology and paralogy in sequence analysis (Kim et al., 2008) is inapplicable to domains at any level of structural abstraction, which by definition include all domain sequence variants (Murzin et al., 1995). According to definitions of the structural classification of proteins (SCOP), protein domains that belong to the same family can be orthologous to each other. Thus each SCOP family is an orthologus evolutionary unit. It is important to note that SCOP classifications are subject to updates and are revised when new information becomes available for protein domains. Thus it is possible that domains previously grouped into different families are later pooled into a single family (Andreeva et al., 2008). Finally, in some cases, domains classified into different fold groups can also be orthologus, as noted by Cheek et al. (2006).
(6) Evolutionary processes such as convergent evolution and horizontal gene transfer (HGT) can confound phylogenetic analysis leading to erratic interpretations when dealing with molecular sequence data. However, the effect of these processes on molecular structure appears to be very limited (Gough, 2005; Choi and Kim, 2007; Forslund et al., 2008; Yang and Bourne, 2009). Even the lower Pfam hierarchical level of structural organization showed limited influence of HGT (<10%; Choi and Kim, 2007). Phylogenetic and statistical analyses revealed that convergent evolution of domain structures are indeed rare in ToDs and ToPs (e.g., Kim and Caetano-Anollés, 2012).
(7) In evolution, macromolecules grow by accretion of structural or substructural components. For example, phylogenetic analysis of the structure of hundreds of tRNAs showed that the modern L-shaped folded cloverleaf structure of the molecule originated in the acceptor arm (Figure 1B) and gradually added stems to its structural make up (Sun and Caetano-Anollés, 2008). Gradual accretion occurs also in larger ribonucleoprotein ensembles such as the RNase P complex (Sun and Caetano-Anollés, 2010) and the ribosome (Harish and Caetano-Anollés, 2012). Similar evolutionary accretion processes drive the evolution of protein molecules and complexes (Gabaldon et al., 2005). For example, a ToD describing the evolution of aminoacyl-tRNA synthetase (aaRS) enzymes at FF level of structural abstraction in SCOP shows gradual discovery of catalytic, editing, trans-editing, anticodon-binding, and accessory domains (Figure 1C). The catalytic domains of class I (c.26.1.1) and class II (d.104.11) aaRSs appear first in evolution closely followed by editing (e.g., the editing FF of ValRS, IleRS, and LeuRS, b.51.1.1), and major anticodon-binding domains (a.27.1.1 and c.51.1.1). While these domains are widely distributed in the proteomes (Nasir et al., 2012), studies show that many accessory domains appearing late in evolution are specific to group of lineages (e.g., many are specific to bacteria). For example, ProRS enzymes that aminoacylate tRNA^Pro^ with proline generally harbor three domains with ages that span two billion years (Gy) of evolution (Figure 1D). In the absence of advanced evolutionary models (Cordoñer and Fares, 2008), sequence analysis fails to take into consideration the historical relationships and evolutionary heterogeneities that exist between domains and the subsets of sequence sites that defines them. In contrast, the study of molecular domains is impervious to the history of domain make up, since the feature that is studied is by definition the domain, the entire molecular module.
(8) ToDs and ToPs are appropriately based on a historical analysis of molecular units of evolution, function, and structure, the protein domains (Murzin et al., 1995). In contrast, ToGs generally consider that genes are the evolutionary units. However, a substantial number of genes code for proteins that have multiple domains (55% in Archaea, 72% in Bacteria, and 84% in Eukarya; Wang and Caetano-Anollés, 2009), each of which contributes confounding histories to phylogenetic reconstruction. More importantly, domains in multidomain proteins are known to gain, loose, and rearrange their domain complement as proteomes evolve (e.g., Moore and Bornberg-Bauer, 2012) Thus, multidomain proteins such as ProRS (Figure 1D) represent evolutionary patchworks that need to be sorted out in sequence analyses. Although, SCOP definitions of protein domains are considered gold standard, SCOP is not completely unbiased. Recently, improvements to the “multidomain” class of protein domains in SCOP have been suggested (Majumdar et al., 2009).
(9) Mutation saturation destroys phylogenetic signal in sequences, a serious problem affecting the validity of deep phylogenetic inference (Sober and Steel, 2002). This problem does not apply to domain abundance, which increases with time, and in doing so enhances deep phylogenetic signal (Wang et al., 2011). The ToD of aaRS domains for example is better supported at its base (Figure 1C). In contrast, ToGs have branches that are best supported when they are not deeply seated in the trees. This poses limitations in the interpretation of phylogenies, especially as these are related to the “tree of life.”
(10) Finally, the most fundamental principle of phylogenetic analysis, character independence, states that each character must serve as an independent hypothesis of evolution (Kluge and Farris, 1969). Violation of character independence is serious and results in phylogenies that do not reflect true evolutionary history (Huelsenbeck and Nielsen, 1999). Molecular structure is defined by interactions between nucleotide sites in a protein sequence (Anisimova et al., 2010). Site co-evolution also results from inter- and intramolecular interactions, functional constraints, and stochastic behavior (Cordoñer and Fares, 2008). For example, aaRS enzymes co-evolve with cognate tRNA molecules and they recognize each other (Figures 1C,D). These mere facts violate character independence of sequence analysis, especially when ToGs include sequences with structures that are divergent. In contrast, ToDs and ToPs are free from this important limitation as long as individual domains (orthologous according to for example SCOP definition) or proteomes, respectively, do not co-evolve with each other (except in cases of symbiosis or parasitism).

ToDs, ToFs, and ToPs are however relatively new to the arsenal of evolutionary bioinformatic approaches and have not been widely used. They are also subject to limitations in structural and functional assignments and the computational demands of sequence-profile or more sensitive profile-profile comparisons [see Nasir et al. (2011) for further discussion]. Their many benefits however outweigh the complexities of dealing with structure and the complex link to function. ToDs and ToFs in particular have considerable potential as these phylogenies provide global and deep views of molecular evolution that are unprecedented. Our experience has shown they have considerable explanatory power and can dissect the evolutionary rise of modern biochemistry (Caetano-Anollés et al., 2012).
